# High Dose Intramuscular Vitamin D3 Supplementation Impacts the Gut Microbiota of Patients With *Clostridioides Difficile* Infection

**DOI:** 10.3389/fcimb.2022.904987

**Published:** 2022-06-06

**Authors:** Sang Hoon Lee, Han-Ki Park, Chang Don Kang, Dae Hee Choi, Sung Chul Park, Jin Myung Park, Seung-Joo Nam, Gi Bong Chae, Kyoung yul Lee, Hyunseok Cho, Sung Joon Lee

**Affiliations:** ^1^ Department of Internal Medicine, Kangwon National University Hospital, Kangwon National University School of Medicine, Chuncheon, South Korea; ^2^ Division of Allergy and Clinical Immunology, Department of Internal Medicine, School of Medicine, Kyungpook National University Chilgok Hospital, Kyungpook National University, Daegu, South Korea; ^3^ Department of Surgery, Kangwon National University Hospital, Kangwon NationalUniversity School of Medicine, Chuncheon, South Korea; ^4^ Department of Pathology, Kangwon National University School of Medicine, Chuncheon, South Korea; ^5^ Department of Hospital Medicine, Kangwon National University School of Medicine, Chuncheon, South Korea

**Keywords:** clostridioides difficile infection, vitamin D, microbiota, cholecalciferol, bifidobacteriaceae, christensenellaceae

## Abstract

**Background and Aim:**

Current therapeutic strategies for *Clostridioides difficile* infections (CDI), including oral vancomycin, metronidazole and fecal microbial transplantation, have limited efficacy and treatment failure may occur in as many as one- third of cases. Recent studies have reported that lower concentrations of 25-hydroxyvitamin D are associated with CDI severity and recurrence. However, there have been no studies on microbiota composition after the administration of vitamin D in patients with CDI. Therefore, our study aimed to compare the microbiota composition between the two groups, including eight CDI-positive patients with vitamin D supplementation and ten CDI-positive patients without vitamin D supplementation by using 16S rRNA microbial profiling.

**Methods:**

Twenty subjects were enrolled in this prospective randomized controlled study. One subject dropped out due to lack of contact with the guardian after discharge and one subject dropped out due to withdrawal of consent. Thus, 18 patients with CDI and vitamin D insufficiency (vitamin D level < 17 ng/mL) were divided into two groups: CDI with vitamin D supplementation (n = 8) and CDI without vitamin D supplementation (control: n = 10). Subjects with vitamin D insufficiency were randomized to receive 200,000 IU intramuscular cholecalciferol whereas patients in the control group received only oral vancomycin. Stool samples were obtained twice before vancomycin was administered and eight weeks after treatment; the V3-V4 16S rRNA metagenomic sequencing was performed using EzBioCloud.

**Results:**

The alpha diversity of the gut microbiota in the recovery state was significantly higher than that in the CDI state. Analysis of bacterial relative abundance showed significantly lower *Proteobacteria* and higher *Lachnospiraceae*, *Ruminococcaceae*, *Akkermansiaceae*, and *Bifidobacteriaceae* in the recovery state. When comparing the control and vitamin D treatment groups after eight weeks, increase in alpha diversity and, abundance of *Lachnospiraceae*, and *Ruminococcaceae* exhibited the same trend in both groups. A significant increase in *Bifidobacteriaceae* and *Christensenellaceae* was observed in the vitamin D group; *Proteobacteria* abundance was significantly lower in the vitamin D treatment group after eight weeks than that in the control group.

**Conclusion:**

Our study confirmed that the increase in the abundance of beneficial bacteria such as *Bifidobacteriaceae*, and *Christensenellaceae* were prominently evident during recovery after administration of a high dose of cholecalciferol. These findings indicate that vitamin D administration may be useful in patients with CDI, and further studies with larger sample sizes are required.

## Introduction

*Clostridioides difficile* is a spore-forming, and toxin-producing, gram-positive anaerobic bacterium. *C. difficile* infection (CDI) is caused by the colonization of *C. difficile* due to changes in the composition of the normal intestinal flora of hospitalized patients receiving antibiotics (Guh and Kutty, 2018). CDI is one of the most common causes of nosocomial infections, and its incidence and mortality rates are increasing worldwide (Guh et al., 2020). In addition, antibiotic use is associated with the recurrence and emergence of antibiotic-resistant bacteria. Current therapies using oral vancomycin or metronidazole are inappropriate for treating intractable severe CDI and preventing recurrent CDI (Surawicz and Alexander, 2011). In recent years, attention has been focused on treatments for the preservation and restoration of intestinal flora and the optimization of the immune response to CDI (Johnson et al., 2021).

Over the past several years, experimental studies have reported the association of vitamin and trace element deficiencies with systemic inflammation and multi-organ failure (Holick, 2007). In particular, vitamin D is involved in the maintenance of bone growth, calcium and phosphorus metabolism, and immune system functions (Chang and Lee 2019). Vitamin D-related epidemiological studies have also reported that vitamin D deficiency increases the risk of systemic infection and is associated with a poor disease course and greater disease activity in patients with chronic inflammatory diseases (Jørgensen et al., 2010; Kempker and Martin, 2013). Recent studies have provided evidence that lower concentrations of 25-hydroxyvitamin D [25(OH)D] (vitamin D level < 20 ng/mL) are associated with CDI severity and recurrence (Sahay and Ananthakrishnan, 2014; Abdelfatah et al., 2015). In another study, vitamin D protect against CDI by restoring melanocyte inducing transcription factor expression and lysosomal function in mice (Chan et al., 2022). However, to date, there have been no studies on changes in microbiota composition after the administration of vitamin D in patients with CDI.

In this prospective observational study, we aimed to compare the microbiota composition by conducting 16S rRNA microbial profiling of two groups: CDI-positive with vitamin D supplementation and CDI-positive without vitamin D supplementation.

## Materials and Methods

### Study Population

**Figure 1:**
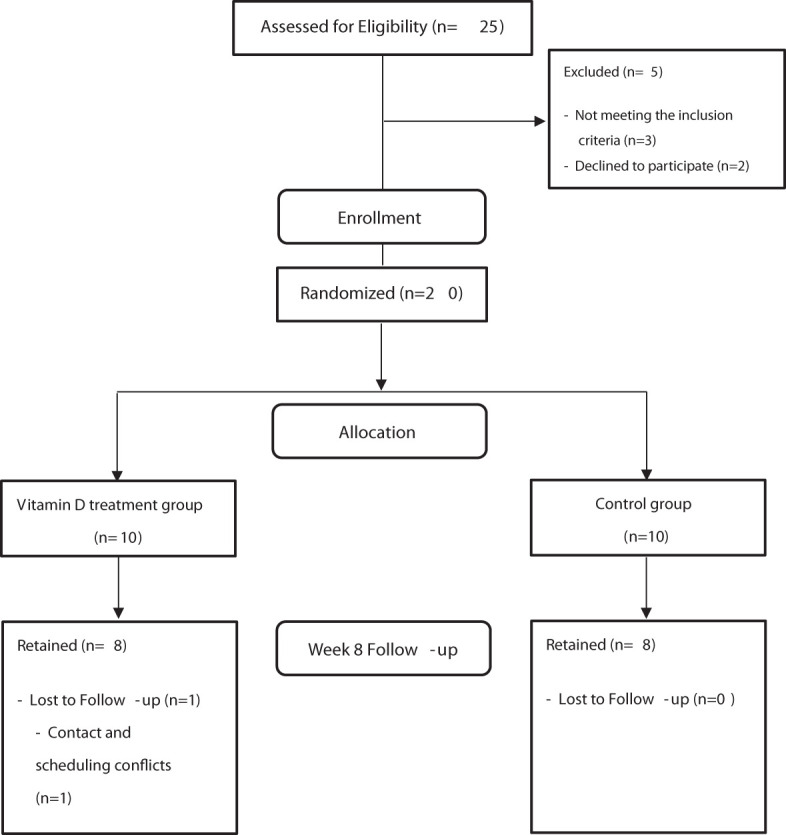
Schematic diagram showing the recruitment process.

This was a prospective, randomized, controlled, and interventional pilot study on 20 patients diagnosed with CDI (defined as ≥3 loose stools in 24 hours without any other cause and a positive glutamate dehydrogenase antigen test, positive Toxin A and Toxin B test or positive polymerase chain reaction) and vitamin D deficiency (serum 25(OH)D levels <17 ng/mL) at Kangwon National University Hospital between October 2019 and June 2021. Twenty subjects were randomly classified into two groups. Exclusion criteria included the use of immunosuppressants, pregnancy or plans to become pregnant in the next 3 months, disorders associated with hypercalcemia, current hypercalcemia (10.8 mg/dL albumin-corrected serum calcium. or 5.2 mg/dL ionized calcium), history of nephrolithiasis, chronic kidney disease worse than stage III, current substantial hepatic dysfunction (2.5 mg/dL total bilirubin, 1.0 mg/dL, direct bilirubin), use of probiotics (within four weeks from the date of CDI diagnosis), and inflammatory bowel disease. The study was approved by the Institutional Review Board of Kangwon National University Hospital (KNUH B-2019-04-005-011). Written informed consent was obtained from all the patients. This research was registered at the Clinical Research Information Service (identifier KCT0004335). Patients in the vitamin D treatment group were administered high-dose vitamin D3 (200,000 IU) *via* intramuscular injection once and were treated with oral vancomycin (125 mg, qid, for 14 days), whereas patients in the control group received only oral vancomycin (**Figure 1**).

### Data Collection

Stool samples were collected from all patients at baseline and at eight weeks after treatment. Stool samples were immediately stored on ice and frozen at −80°C. DNA was extracted from the collected samples using a sterile container, and the composition of the microorganisms was analyzed *via* 16S rRNA sequencing using the extracted DNA.

### DNA Extraction, PCR Amplification and Sequencing

Total DNA was extracted using the Maxwell^®^ RSC PureFood GMO and Authentication Kit (Promega, USA), according to the manufacturer’s instructions. PCR amplification was performed using fusion primers targeting the V3-V4 region of the 16S rRNA gene. For bacterial amplification, the fusion primers 341F (5’-AATGATACGGCGACCACCGAGATCTACAC-XXXXXXXX-TCGTCGGCAGCGTC-AGATGTGTATAAGAGACAG-CCTACGGGNGGCWGCAG-3’; underlined sequence indicates the target region primer) and 805R (5’- CAAGCAGAAGACGGCATACGAGAT-XXXXXXXX-GTCTCGTGGGCTCGG-AGATGTGTATAAGAGACAG-GACTACHVGGGTATCTAATCC-3’) were used. The fusion primers were constructed in the following order: P5 (P7) graft-binding, i5 (i7) index, Nextera consensus, sequencing adaptor, and target region sequence. The amplification conditions were as follow: initial denaturation at 95°C for 3 min, followed by 25 cycles of denaturation at 95°C for 30 s, annealing at 55°C for 30 s, extension at 72°C for 30 s, and a final elongation at 72°C for 5 min. The PCR product was confirmed using 1% agarose gel electrophoresis and visualized using a Gel Doc system (Bio-Rad, Hercules, CA, USA). The amplified products were purified using CleanPCR (CleanNA). Equal concentrations of the purified products were pooled and short fragments (non-target products) were removed using CleanPCR (CleanNA). Quality and product size were assessed on the Bioanalyzer 2100 (Agilent, Palo Alto, CA, USA) using a DNA 7500 chip. Mixed amplicons were pooled and sequencing was performed by Chunlab, Inc. (Seoul, Korea), using the Illumina MiSeq Sequencing system (Illumina, USA) according to the manufacturer’s instructions. Sequence data were deposited in the National Center for Biotechnology Information as Bio Project ID: PRJNA824324.

### Data Analysis Pipeline

The processing of raw reads started with quality check and filtering of low quality (<Q25) reads by Trimmomatic ver. 0.321. After QC pass, paired-end sequence data were merged together using the fastq_mergepairs command of VSEARCH version 2.13.42 with default parameters. Primers were then trimmed using the alignment algorithm of Myers and Miller3 at a similarity cutoff of 0.8. Non-specific amplicons that do not encode 16S rRNA were detected using nhmmer (Wheeler and Eddy, 2013) in the HMMER software package ver. 3.2.1 with hmm profiles. Unique reads were extracted and redundant reads were clustered with the unique reads by the derep_fulllength command of VSEARCH (Rognes et al., 2016). The EzBioCloud 16S rRNA database5 was used for taxonomic assignment using usearch global command of VSEARCH2 followed by more precise pairwise alignment3. Chimeric reads were filtered on reads with <97% similarity by reference based chimeric detection using the UCHIME algorithm6 and the non-chimeric 16S rRNA database from EzBioCloud. After chimeric filtering, reads that are not identified at the species level (with <97% similarity) in the EzBioCloud database were compiled and cluster_fast command2 was used to perform *de-novo* clustering to generate additional Operational taxonomic units. Finally, OTUs with single reads (singletons) were omitted from further analyses. The secondary analysis which includes diversity calculation and biomarker discovery was conducted using in-house programs of Chunlab, Inc (Seoul, South Korea). Alpha diversity information was confirmed through the Chao1 value, and Shannon. The relationship between samples was visualized through principal coordinate analysis (PCoA) using the Bray-Curtis dissimilarity and beta diversity distances were calculated using the Bray-Curtis dissimilarity index.

Linear discriminant Effect Size (LEfSe) analysis was performed to identify bacteria that were significantly different; the degree of difference was expressed as a linear discriminant analysis (LDA) score with α = 0.05 and LDA score threshold-2. At this time, 1% or more of the genus level was analyzed. All analyses mentioned above were performed in the EzBioCloud 16S-based MTP, which is a Chunlab bioinformatics cloud platform.

### Statistical Analysis

Continuous variables were analyzed using the Mann–Whitney U test. Fisher’s exact test was used to compare the categorical variables between the control and experimental group. Paired data were analyzed using the paired Wilcoxon signed-rank test. In the statistical analysis, continuous variables were analyzed using t-test when P ≥ 0.05 and the Shapiro-Wilk and the Mann-Whitney U tests when P < 0.05. Statistical analyses were performed using SPSS for Windows (version 19.0; IBM Co., Armonk, NY, USA). Statistical significance was set at P < 0.05. GraphPad Prism 9.0 software (GraphPad Inc., San Diego, CA, USA) was used to generate the graphs.

## Results

### Demographic and Clinical Characteristics

The demographic and clinical characteristics of the patients are summarized in **Table 1**. Twenty subjects who met the inclusion criteria were enrolled; however, one subject dropped out due to no contact with the guardian after discharge, and one subject dropped out due to withdrawal of consent. Thus, a total of 18 subjects was analyzed. No differences in age, sex, BMI, presence of hypertension, presence of diabetes, cardiovascular disease, liver disease, chronic kidney disease, or chronic respiratory disease were observed between the vitamin D treatment and control groups. In addition, no statistically significant difference between the two groups in any of the tests performed were observed, except for blood urea nitrogen in the hematological examination (**Table 1**).

**Table 1:** Demographic and clinical characteristics of study population (N=18).

	Control (n = 10)	Experiment (n = 8)	P-value
**Age(yr)**	76.9 ± 13.3	71.5 ± 21.9	0.573
**Sex(M:F)**	5 vs 5	4 vs 4	1
**BMI(kg/m2)**	20.9 ± 2.9	20.4 ± 4.6	0.46
**Hypertension - no.(%)**	5(50)	4(50)	1
**Diabetes mellitus- no.(%)**	2(20)	2(25)	1
**Caridiac_disease- no.(%)**	2(20)	0(0)	0.477
**Liver_disaeas- no.(%)**	1(10)	0(0)	1
**Chronic_renal_disease** **- no.(%)**	3(30)	0(0)	0.216
**Chronic_pulmonary_disease** **- no.(%)**	2(20)	1(12.5)	1
**WBC(/ul)**	17260 ± 11931	11675 ± 4062	0.36
**Neutrophil_count(%)**	82.3 ± 9.0	74.2 ± 12.7	0.146
**Hemoglobin(g/dL)**	11.5 ± 1.5	11.1 ± 1.6	0.633
**Platelet_count(/ul)**	211 ± 116	289 ± 104	0.237
**ESR(mm/hr)**	17.7 ± 32.6	45.2 ± 17.5	0.065
**CRP(mg/dL)**	9.9 ± 6.5	9.0 ± 6.0	0.965
**BUN(mg/dL)**	34.1 ± 34.4	12.8 ± 5.6	0.016
**Creatine(mg/dL)**	1.5 ± 1.3	0.7 ± 0.2	0.173
**total_bilirubin(mg/dL)**	0.7 ± 0.2	0.7 ± 0.3	0.762
**Albumin(g/dL)**	3.2 ± 0.8	3.2 ± 0.6	0.829
**Vitamin_D(ng/mL)**	10.1 ± 3.8	9.8 ± 4.4	0.897

BMI, body mass index; WBC, white blood cell count; ESR, erythrocyte sedimentation rate; CRP, C-reactive protein; BUN, blood urea nitrogen.

### Changes in the Gut Microbiota at the Time of CDI and Recovery After Eight Weeks

**Figure 2:**
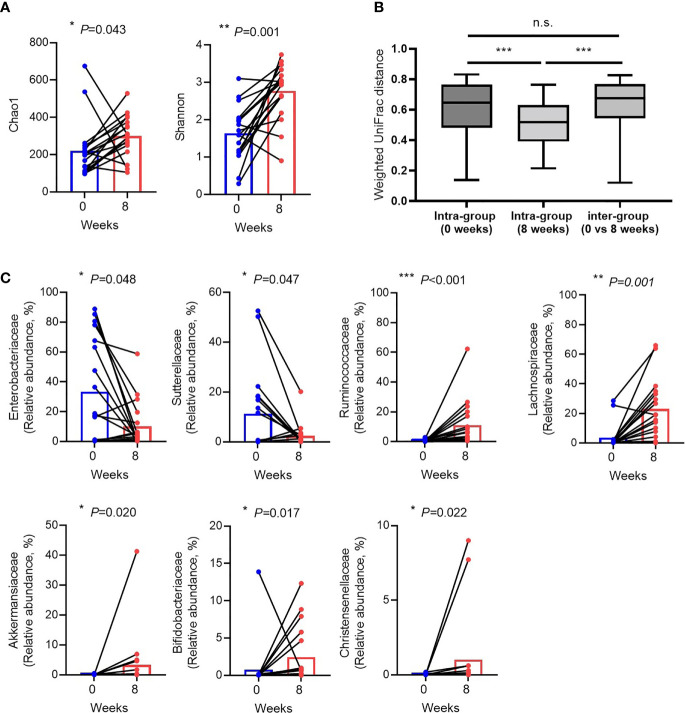
Changes in the gut microbiota between *Clostridioides* difficile infection and recovery after eight weeks. **(A)** Alpha diversity. **(B)** Weighted UniFrac distance. **(C)** Relative abundances of individual bacteria that were significantly different between *Clostridioides* difficile infection and recovery. Blue dots indicate C. difficile infection state (0 week), red dots indicate recovery after eight weeks, and black lines represent changes in the same patient. n.s, not significant; *P < 0.05, **P < 0.01 and ***P < 0.001 (Wilcoxon signed rank test and Mann-Whitney U test).

Alpha diversity, particularly in the Shannon index was increased (**Figure 2A**). The beta diversity using principal coordinate analysis was higher in terms of weighted UniFrac distance in CDI than in the recovery state (**Figure 2B**). When looking at the changes in individual species, the abundance of *Proteobacteria* (*Enterobacteriaceae* and *Sutterellaceae*) and *Enterococcaceae*, which are generally known to increase during CDI or antibiotic treatment, increased during CDI compared to those after recovery. In particular, a significant reduction in *Proteobacteria* (47.61 ± 37.20% in CDI vs. 13.63 ± 16.79% in recovery, P = 0.002) was evident during the recovery period. Conversely, the numbers of commensal bacteria and beneficial strains increased during the recovery period. A statistically significant increase in the abundance of short-chain fatty acid (SCFA)-producing clostridia such as *Lachnospiraceae* (3.50 ± 8.17% in CDI vs. 22.92 ± 19.66% in recovery, *P* = 0.001 and *Ruminococcaceae* (0.49 ± 0.80% in CDI vs. 11.12 ± 15.27% in recovery, *P* < 0.001) was observed (**Figure 1C**). Beneficial strains such as *Akkermanssiaceae* (0.03 ± 0.13% in CDI vs. 3.35 ± 9.71% in recovery, *P* = 0.020), *Bifidobacteriaceae* (0.79 ± 3.27% in CDI vs. 2.44 ± 3.79% in recovery, *P* = 0.017) and *Christensenellaceae* (0.13 ± 0.04% in CDI vs. 1.02 ± 2.69% in recovery, *P* = 0.022) also showed increased abundance. The decrease in *Proteobacteria* abundance and the increase in *Lachnospiraceae* and *Ruminococcaceae* abundance during the recovery period showed a statistically strong association (**Figure 2C**).

### Changes in the Gut Microbiota in the Control and Vitamin D Treatment Groups

**Figure 3:**
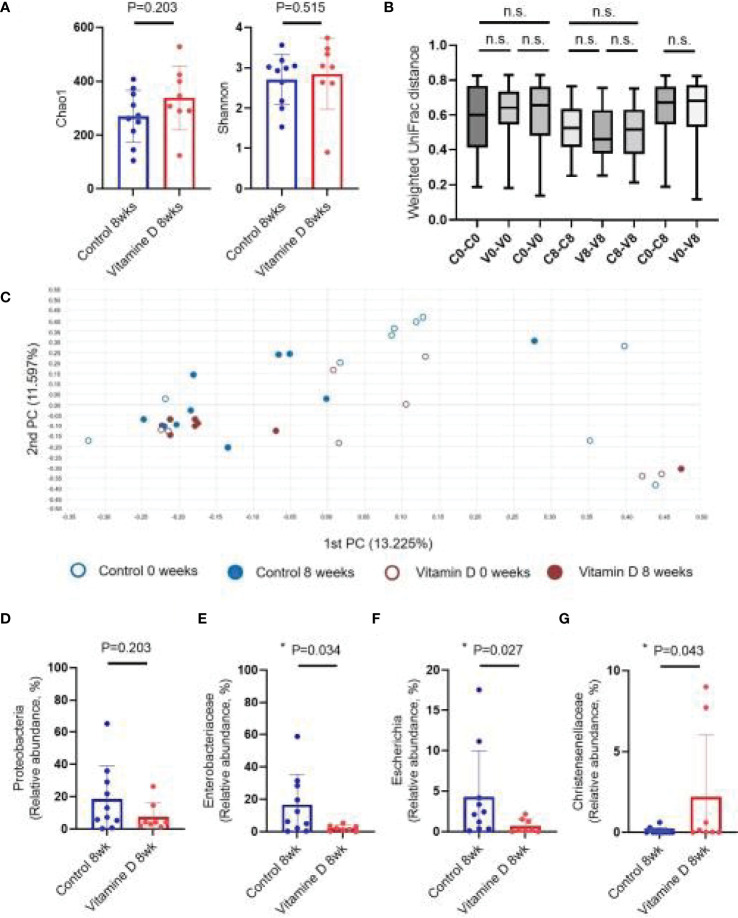
Effect of vitamin D supplementation on the gut microbiome. **(A)** Alpha diversity. **(B)** Weighted UniFrac distance. **(C)** Principal coordinates analysis (Bray–Curtis dissimilarity). **(D)** Relative abundances of *Proteobacteria*. **(E)** Relative abundances of *Enterobacteriaceae*. **(F)** Relative abundances of Escherichia. **(G)** Relative abundances of *Christensenellaceae*. n.s, not significant; *P < 0.05 (Wilcoxon signed rank test and Mann-Whitney U test).

No significant difference in the alpha diversity between the vitamin D treatment and control groups were observed (**Figure 3A**). Beta diversity was not significantly different between the vitamin D treatment and control groups (**Figures 3B**, **3C**). In addition, no statistically significant differences at the phylum level were observed between the two groups (**Supplementary figure 1**).

With respect to individual taxon, the abundance of *Proteobacteria*, which significantly increased during CDI, showed a downward trend in the vitamin D treatment group, but the difference was not statistically significant. At the family and genus levels, the vitamin D treatment group showed a significant decrease in *Enterobacteriaceae* (16.50 ± 18.69% in CDI vs. 2.16 ± 1.88% in recovery, P = 0.034) and *Escherichia* (4.29 ± 5.68% in CDI vs. 0.69 ± 0.85% in recovery, P = 0.027) compared to those in the control group (**Figures 3D–F** and **Supplementary Figure 2**). Furthermore, the abundance of *Christensenellaceae *and* Sutterellaceae* was higher in the vitamin D treatment group. However, the vitamin D treatment group decreased more effectively than CDI and recovery groups (**Figure 3G**, **Supplementary Figure 2** and **Supplementary Figure 3**).

**Figure 4:**
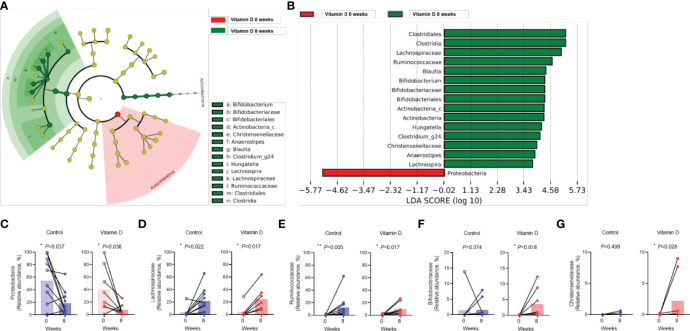
Effect of vitamin D supplementation on the individual gut taxa. A, B. Cladogram **(A)** and linear discriminant analysis scores **(B)** using LEfSe analysis of the fecal microbiota in *Clostridioides* difficile infection versus recovery after eight weeks in the vitamin D treatment group. **(C–G)**. Differences in the abundance of individual bacteria between the control group and vitamin **(D)** supplement group. **(C)** *Proteobacteria*. **(D)** *Lachnospiraceae*. **(E)** *Ruminococcaceae*. **(F)** *Bifidobacteriaceae*. **(G)** *Christensenellaceae*. *P < 0.05, **P < 0.01 (Wilcoxon signed rank test).

The increase in *Lachnospiraceae*, *Ruminococcaceae*, *Bifidobacteriaceae*, and *Christensenellaceae* were evident in the vitamin D treatment group, whereas the abundance of *Proteobacteria* decreased (**Figures 4A, B**). Changes in *Proteobacteria*, *Lachnospiraceae*, and *Ruminococcaceae* abundance were also observed in both the vitamin D treatment group and the control group (**Figures 4C–E**). However, the increase in *Bifidobacteriaceae* and *Christensenellaceae* abundance was more prominent in the vitamin D treatment group than that in the control group (**Figures 4F, G**).

## Discussion

This study investigated the changes in the gut microbiota between CDI and recovery in patients with and without vitamin D supplementation. Specifically, we found a significant increase in the abundance of *Proteobacteria* during CDI, and an increase in the abundance of *Lachnospiraceae*, *Ruminococcaceae*, *Akkermansiaceae*, *Bifidobacteriaceae*, and *Christensenellaceae* after recovery. The vitamin D treatment group showed a significant increase in the abundance of beneficial bacteria, such as *Bifidobacteriaceae* and *Christensenellaceae*, and the abundance of *Enterobacteriaceae* in the recovery state was significantly lower than that in the group without vitamin D treatment.

To date, the overall incidence of CDI and severe or fulminant CDI have increased, in association with an increased infection by highly virulent strains, such as NAP1/BI/027, and the increased use of antibiotics, anticancer drugs, and gastric acid inhibitors (Pépin et al., 2004; Ricciardi et al., 2007). Another problem is the increased frequency of CDI recurrence that may be caused by the reactivation of previous bacteria or re-infection with new bacteria. According to previous studies, up to 25% of patients experience recurrent CDI within 30 days of treatment (Kelly, 2012). The risk of subsequent recurrence in patients was 45% (McFarland et al., 2002). In the treatment of severe, refractory, or recurrent CDI, vancomycin has shown limitations in enhancing the effectiveness of treating or preventing recurrence. Recently, many studies have investigated fecal microbiota transplantation (FMT) as a treatment for CDI. Conventional CDI treatment with vancomycin disrupts the balance of normal colonic flora and weakens the resistance of normal flora to other proliferating bacteria. In FMT, this imbalance is restored by transplanting donor feces containing normal flora into patients with CDI (Kim, 2012). However, FMT has procedural complications and risks the transmission of infectious agents (Reumkens et al., 2016; Zellmer et al., 2021).

Vitamin D plays an important role in the maintenance of bone mineral density and has an important influence on the immune system, including the modulation of antiviral and antibacterial inflammatory immune responses (Chang and Lee 2019). Most studies emphasize maintaining vitamin D levels above 30 ng/ml to prevent osteoporosis and rickets (Dawson-Hughes et al., 2010; American Geriatrics Society Workgroup on Vitamin D Supplementation for Older Adults Workgroup on Vitamin, 2014). It also has an important influence on the immune system (Taha et al., 2021),. Vitamin D induces cathelicidin production, which can directly kill viruses and bacteria or bind to endotoxins (Liu et al., 2006). Studies on vitamin D deficiency and supplementation in inflammatory bowel disease (IBD) and inherited disorders, such as cystic fibrosis, have been published. When vitamin D was administered daily for one month to patients with IBD and vitamin D deficiency, a negative relationship between vitamin D levels and C-reactive protein was found (Jun et al., 2019). Kanhere et al. (2018) reported that in patients with cystic fibrosis and vitamin D deficiency, an imbalance in the intestinal microflora was observed, which improved when high doses of vitamin D were administered. Abdelfatah et al. (2015) suggested a significant relationship between vitamin D levels and CDI severity. In the present study, we investigated the effect of vitamin D by applying a strict standard of less than 17 ng/mL, which is lower than the vitamin D deficiency standard (Taha et al., 2021). When vitamin D3 (200,000 IU) was administered to patients with CDI at the initial stage of infection, vitamin D deficiency was quickly corrected, but the difference was not statistically significant compared to the control group in relieving microbiota dysbiosis.

*Firmicutes* and *Bacteroidetes* dominate the normal intestinal environment, whereas *Proteobacteria*, *Actinobacteria*, and *Verrucomicrobia* are less abundant (Backhed et al., 2005). An increased prevalence of *Proteobacteria* is a marker of an imbalance in the taxonomic composition of the gut microbiota and a potential diagnostic criterion for disease (Shin et al., 2015). In particular, an increase in *Proteobacteria* is evident when the gut microbiota changes, and metabolic homeostasis is disrupted by the use of antibiotics (Zarrinpar et al., 2018). In CDI, *Proteobacteria* and *Enterococcus* strains are the major strains that increase during dysbiosis owing to changes in the gut microbiota (Samarkos et al., 2018; Kim et al., 2020). *Proteobacteria* induces epithelial dysfunction and exacerbates intestinal inflammation, and the association between CDI and IBD is well known (Litvak et al., 2017; Caruso et al., 2020). In contrast, *Bacteroides*, *Prevotella*, and *Clostridiales* are commensal bacteria, and enterotyping is performed depending on which strain is dominant (Arumugam et al., 2011). Among them, SCFA-producing *Clostridium*, as well as *Lachnospiraceae* and *Ruminococcaceae*, play an important role in the anti-inflammatory action of intestinal immunity by increasing T reg activity (Guo et al., 2020; Park et al., 2020). In a previous study, patients with CDI showed a decrease in *Lachnospiraceae* and *Ruminococcaceae* (Antharam et al., 2013). In the present study, the increase in *Proteobacteria* abundance after CDI was substantial. Furthermore, during the recovery process, the decrease in *Proteobacteria* abundance and increase in the abundance of *Lachnospiraceae* and *Ruminococcaceae*, which are important for the secretion of metabolites such as SCFA with anti-inflammatory effects, particularly among normal flora were prominent. Therefore, methods that reduce *Proteobacteria* abundance and increase the abundance of commensal *Clostridiales*, such as *Lachnospiraceae* and *Ruminococcaceae*, could be used to treat CDI. *Akkermansia* is also a candidate probiotic that has recently received attention for its role in metabolic and systemic diseases (Plovier et al., 2017; Michalovich et al., 2019).

In addition, we observed that the abundance of *Bifidobacteriaceae* and *Christensenellaceae* significantly increased with vitamin D supplementation. *Bifidobacterium*, a well-known probiotic, is effective against inflammatory diseases by reducing inflammatory substances including cytokines, protecting the intestinal epithelial barrier, and balancing the gut microbiota (Konieczna et al., 2012). The anti-inflammatory effects of *Bifidobacterium* in animal dextran sodium sulfate models and human IBD have also been consistently reported (Singh et al., 2020; Yao et al., 2021). In a recent study involving vitamin D supplementation in healthy controls, an increase in *Bifidobacterium* abundance was observed (Meng et al., 2020). Previous studies have reported that *Bifidobacteriaceae* reduces CDI through anti-inflammatory effects (Skraban et al., 2013; Singh et al., 2020). Meanwhile, the function of *Christensenellaceae* is relatively less known, but it is a commensal bacterium in the human gut that plays an important role in human health (Waters and Ley, 2019). Further research is needed on the effects of *Bifidobacteriaceae* and *Christensenellaceae* on CDI and their relationship with vitamin D. Fifth, it was not possible to evaluate whether there were intestinal changes in increased intestinal permeability following high-dose vitamin D supplementation. In our first study plan, we tried to perform biopsy by performing sigmoidoscopy at the time of initial diagnosis and 8 weeks after treatment for CDI. Through this biopsy, it was attempted to determine whether there were any changes in the tissues depending on whether or not high-dose vitamin D was administered, but it was not possible to obtain the consent of the patients and guardians.

This study has several limitations. First, since this was a pilot study, the sample size was small, which may be statistically underpowered. To overcome this limitation, we included a control group. Second, selection bias may be possible because only patients with CDI with a vitamin D level of less than 17 ng/mL were enrolled in our study. Third, other parameters such as diet were not evaluated. Further investigations, including dietary patterns and other predisposing factors that could affect the microbiome, would help deepen our understanding of the relationship between vitamin D and the microbiome. Fourth, the short-term effects of vitamin D supplementation could not be assessed. In this study, the control group also showed a significant change after eight weeks compared to the CDI group. Therefore, to observe the effects of vitamin D, additional analysis over a short period of one–two weeks may be necessary.

In conclusion, our study is the first to identify changes in the gut microbiota upon administration of high-dose vitamin D to patients with CDI and show that the administration of a high dose of cholecalciferol may play an adjuvant role in the treatment of CDI for the first time. Furthermore, our study confirmed that the increase in *Christensenellaceae* and *Bifidobacteriaceae* abundance was enhanced in vitamin D-deficient patients with CDI recovery after the administration of a high dose of cholecalciferol. Physicians should consider the potential role of vitamin D as replacement therapy in patients with CDI. These findings require further evaluation in a larger, multicenter study.

## Data Availability Statement

The datasets presented in this study can be found in online repositories. The name of the repository and accession number can be found below: NCBI; PRJNA824324.

## Ethics Statement

The studies involving human participants were reviewed and approved by the Institutional Review Board of Kangwon National University Hospital. The patients/participants provided their written informed consent to participate in this study.

## Author Contributions

SL and H-KP: design of the study. SL, CK, DC and JP: data acquisition. H-KP, KL and HC: data analysis, and interpretation. S-JN, SP and GC: drafted the article and critically revised the manuscript. SL: gave final approval for the version to be submitted. All authors reviewed and approved the final version of the manuscript.

## Funding

This study received funding from 2019 Hanmi Pharm. Co. The funder was not involved in the study design, collection, analysis, interpretation of data, the writing of this article or the decision to submit it for publication. All authors declare no other competing interests.

## Conflict of Interest

The authors declare that the research was conducted in the absence of any commercial or financial relationships that could be construed as a potential conflict of interest.

## Publisher’s Note

All claims expressed in this article are solely those of the authors and do not necessarily represent those of their affiliated organizations, or those of the publisher, the editors and the reviewers. Any product that may be evaluated in this article, or claim that may be made by its manufacturer, is not guaranteed or endorsed by the publisher.
