# Supplementary figures and images for: High Dose Intramuscular Vitamin D3 Supplementation Impacts the Gut Microbiota of Patients With Clostridioides Difficile Infection
Source: Front Cell Infect Microbiol. 2022 Jun 6;12:904987. doi: 10.3389/fcimb.2022.904987 (PMC9239168; doi:10.3389/fcimb.2022.904987)

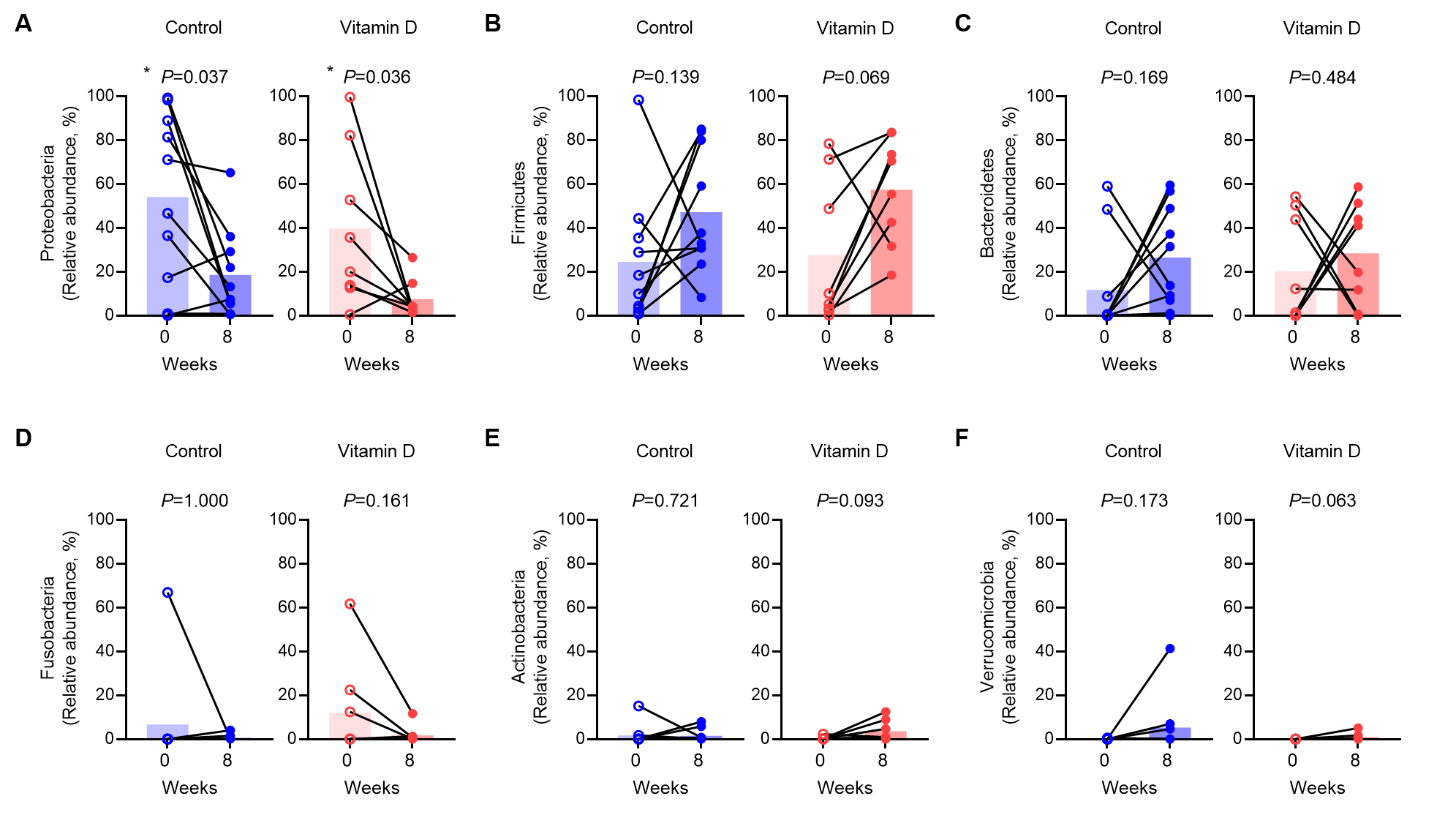

Supplement: Supplementary file 1 [file Image_1.png]

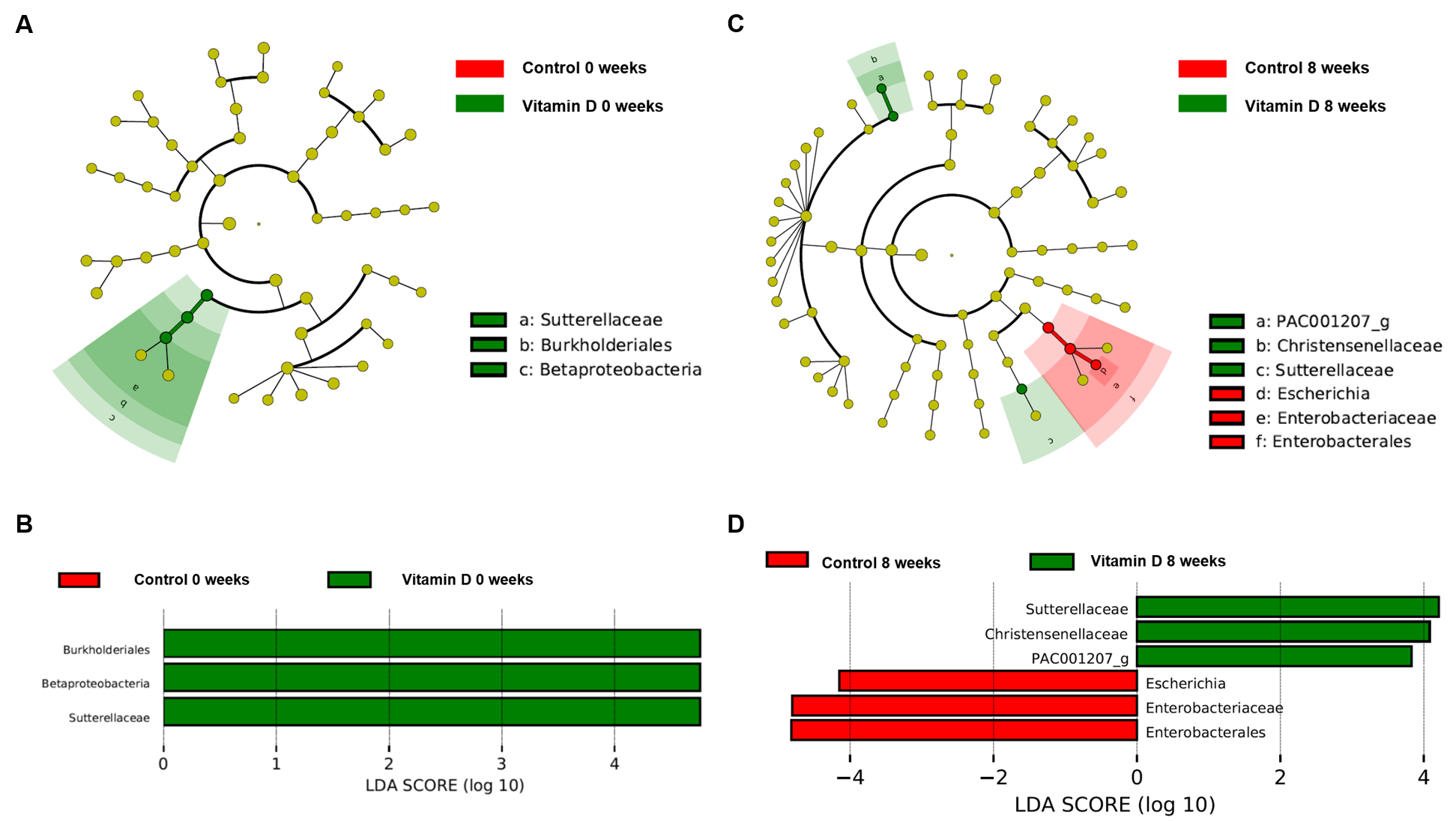

Supplement: Supplementary file 2 [file Image_2.png]

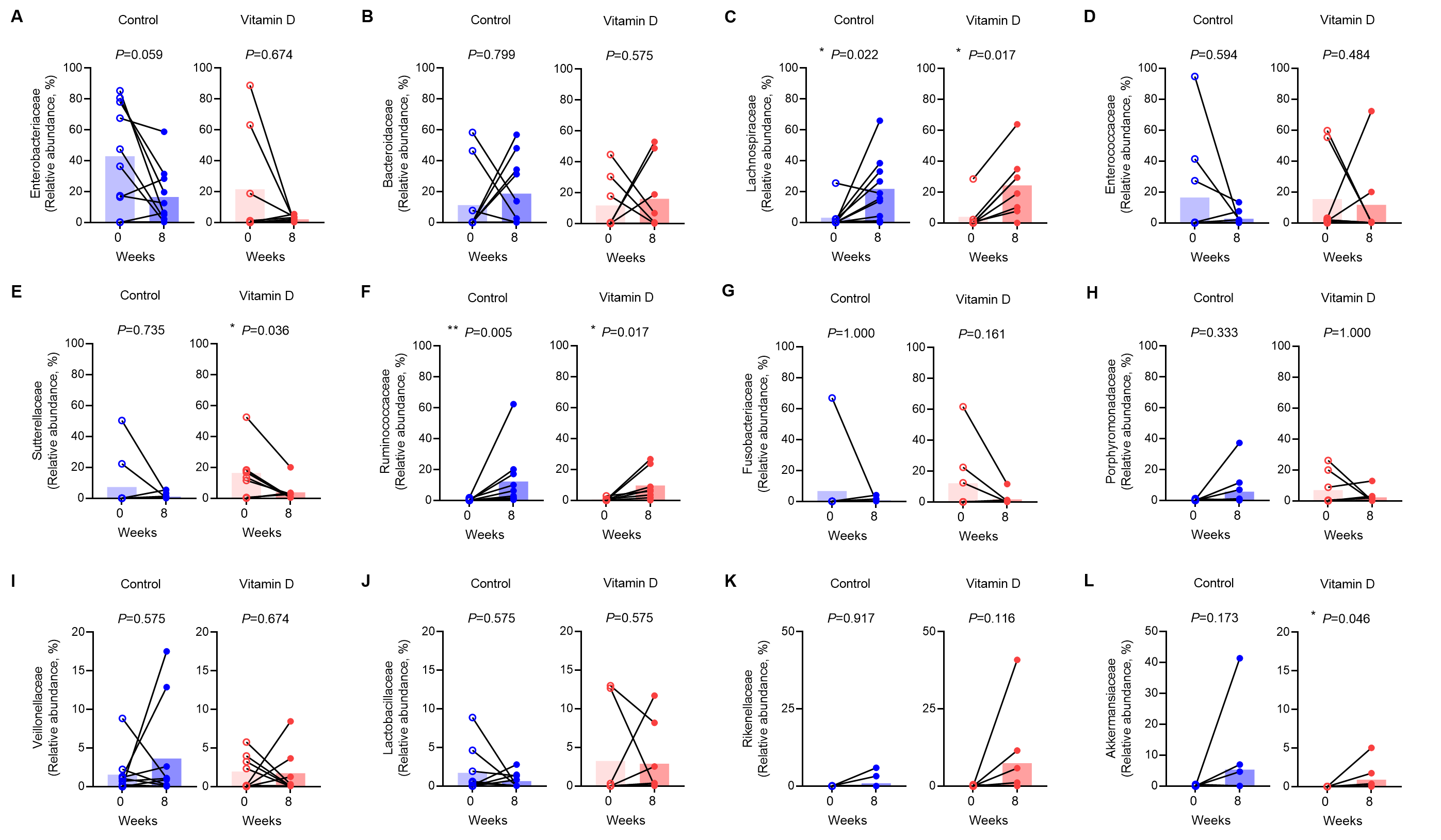

Supplement: Supplementary file 3 [file Image_3.png]
